# Clinical Description and Sociodemographic Profile of Individuals with Cybersex Addiction in Long-Term Therapeutic Support Programs

**DOI:** 10.3390/healthcare14121718

**Published:** 2026-06-15

**Authors:** Luís Lorente-Corral, David Sancho-Cantus, Samuel Asensio, Cristina Cunha-Pérez, Jorge Casaña Mohedo

**Affiliations:** 1 Doctoral Degree School, Catholic University San Vicente Mártir, 46001 Valencia, Spain; luis.lorente@ucv.es; 2 Department of Nursing, Catholic University San Vicente Mártir, 46001 Valencia, Spain; david.sancho@ucv.es (D.S.-C.); jorge.casana@ucv.es (J.C.M.); 3 Nursing & Mental Health Research Group, Faculty of Medicine and Health Sciences, Catholic University San Vicente Mártir, 46001 Valencia, Spain; 4 Department of Biomedical Sciences, Institute of Biomedical Sciences, Faculty of Health Sciences, Cardenal Herrera–CEU University, 46115 Valencia, Spain; 5 SONEV Research Group, Faculty of Medicine and Health Sciences, Catholic University San Vicente Mártir, 46001 Valencia, Spain

**Keywords:** hypersexuality, cybersex addiction, self-esteem, impulse control, therapeutic support

## Abstract

Background/objective: Cybersex addiction and hypersexual behavior represent escalating challenges in global mental health. This study analyzed the sociodemographic and clinical profiles of a cohort of males undergoing treatment, examining the concurrent associations between therapeutic support duration and symptomatic severity. Method: An observational, descriptive, and cross-sectional study was conducted with a predominantly male clinical cohort (*n* = 27; 26 males and 1 female) enrolled in therapeutic support programs. Assessment instruments included the Internet Sex Screening Test (ISST), the Hypersexual Behavior Inventory (HBI), and the Rosenberg Self-Esteem Scale (RSES). Results: Findings revealed a pornography consumption pattern characterized by high intensity and early onset of behavioral addiction. A significant prevalence of low self-esteem was detected (48.1%). Statistical analysis demonstrated that neither age of onset nor self-esteem levels significantly correlated with current disorder severity. Conclusions: A descriptive pattern was identified regarding the duration of therapeutic follow-up, where patients with longer program tenure exhibited lower scores in impulse control difficulties. While early onset and self-esteem function as concurrent clinical characteristics, they do not linearly correlate with current clinical severity. Therapeutic support duration is associated with specific indicators of clinical stability, suggesting the potential utility of long-term nursing-led interventions.

## 1. Introduction

While classic theoretical frameworks traditionally conceptualized addiction through the exclusive lens of substance use, contemporary psychiatric [1] and psychological paradigms recognize behavioral addictions as distinct clinical entities sharing overlapping neurobiological and phenomenological features. Within this spectrum, Compulsive Sexual Behavior Disorder (CSBD), newly classified in the 11th edition of the International Classification of Diseases (ICD-11) in 2022, has emerged as a major global mental health concern. This diagnosis is based on a persistent pattern (minimum 6 months) of failure to control intense sexual impulses, leading to the neglect of personal health, social interests, and occupational responsibilities, persisting even when the satisfaction obtained is minimal or the consequences are severely adverse [2,3].

However, a critical review of the current literature reveals a significant empirical gap. The vast majority of existing studies on cybersex and hypersexuality rely predominantly on cross-sectional surveys deployed in convenience, non-clinical samples, such as university students or community online panels [4,5]. While these studies are valuable for assessing epidemiological prevalence and initial correlates, they fail to capture the complex clinical reality of patients who exhibit severe functional impairment and seek professional treatment [6].

Currently, there is a scarcity of empirical data characterizing authentic clinical cohorts, specifically those engaged in long-term specialized therapeutic support programs. Understanding the sociodemographic configurations, chronological patterns of early exposure, and core psychopathological mechanisms (such as maladaptive coping and self-esteem dynamics) within an established clinical sample is essential. This information is a prerequisite for designing evidence-based, long-term care protocols in mental health nursing, optimized for managing clinical indicators within these complex profiles. Consequently, this study aims to address this gap in the literature by providing a detailed clinical description of individuals undergoing structured therapeutic support for cybersex addiction [7].

### 1.1. Multidimensional Consequences and the Early Onset Controversy

Scientific evidence demonstrates that the recurrent consumption of explicit sexual content carries significant clinical implications across physiological, psychological, and relational domains. However, when evaluating the potential associations of these chronological patterns, a profound debate and conflicting findings emerge within the contemporary literature regarding the true prognostic value of the age of consumption onset [3,5].

On one side of the scientific spectrum, several neurodevelopmental and behavioral models argue that early exposure to highly explicit digital material during critical windows of adolescent development is a significant risk factor, potentially altering reward mechanisms, inducing cognitive desensitization, and relating to a greater severity of hypersexual symptoms in adulthood. Conversely, a growing body of empirical research challenges this deterministic view, demonstrating weak or entirely non-significant direct statistical links between precocious onset and adult psychopathological severity once macro-variables such as moral incongruence, religious background, or general psychological distress are controlled. These conflicting frameworks suggest that the age of first contact might function as a distal, non-linear characteristic rather than a direct clinical indicator of current severity [6,7,8].

This divergence in the literature underscores a critical gap: the lack of clear consensus on how chronological onset co-occurs with core maintenance mechanisms—such as self-esteem dynamics and the deployment of cybersex as a maladaptive coping strategy—within clinical cohorts undergoing active treatment. Consequently, evaluating these conflicting paradigms within a specialized treatment-seeking sample is necessary to clarify current prognostic models and optimize targeted mental health nursing interventions.

### 1.2. The Impact of the Digital Era and High-Speed Broadband Internet

The transition from physical to digital pornography has altered consumption patterns. The development of the Internet and high-speed broadband infrastructure has generalized access through availability, affordability, and anonymity [8]. Between 2004 and 2016, the proportion of online consumers increased by 310% [9]. Portals such as Pornhub reported a growth from 14.7 billion visits in 2013 [10], reaching 47 billion during the 2020 pandemic lockdown [11]. This consumption pattern predominantly affects males within specialized treatment-seeking cohorts, indicating that up to 92.6% of these patients present a daily frequency or higher [12,13,14].

### 1.3. Multidimensional Consequences and the Role of Self-Esteem

Empirical evidence demonstrates that the recurrent consumption of explicit sexual content is associated with physiological dysfunctions, such as premature ejaculation and pornography-induced impotence [15]; psychological symptoms, including depression, sleep disorders, attention deficit hyperactivity disorder, and the erosion of self-concept [16,17]; and relational difficulties, such as distortion of partner expectations, aggressive behaviors, and social isolation [18,19]. The prevalence of low self-esteem in this population is estimated at 48.1% in clinical samples. However, current research suggests that self-esteem functions as a concurrent comorbidity or a consequence of the overall functional impairment caused by the disorder rather than as a direct correlate of symptom severity [20]. The primary driver of deterioration appears to be the utilization of sexual behavior as a maladaptive coping strategy to manage negative emotional states such as frustration, loneliness, or stress [21].

### 1.4. Recovery as a Relational Process and Group Support

The clinical presentation is frequently characterized by intense feelings of shame and fear, which can lead to social isolation and complicate the therapeutic experience [22,23]. Although clinical progress is an individual experience, the literature emphasizes its relational framework [22], identifying social interaction and support groups as modalities where individuals can reconstruct their identity outside of compulsive behavioral patterns. Structured group interventions are recognized as valuable approaches to reduce shame and challenge denial [22,24]. In this context, the duration of retention within therapeutic support programs is associated with specific clinical indicators, such as lower levels of impulse control difficulties [14]. Given the scarcity of research analyzing long-term support programs from a mental health nursing perspective, exploring the sociodemographic and clinical characteristics of these cohorts is warranted. This study evaluates the concurrent associations between the age of consumption onset, self-esteem metrics, and program tenure to provide data that may inform group intervention strategies. Consequently, the primary objective of this study is to outline the clinical characterization and sociodemographic profile of a cohort under long-term specialized therapeutic support for compulsive cybersex behavior. Specifically, this work examines the cross-sectional relationships between chronological indicators, clinical dimensions of hypersexuality, self-esteem levels, and psychological coping mechanisms. To guide this evaluation, the following research hypotheses were formulated:

**H1:**  *The age of onset of pornography consumption will not demonstrate a significant statistical association with the current clinical severity of compulsive cybersex behavior in adulthood.*

**H2:**  *Maladaptive coping strategies, specifically the utilization of sexual behavior as an emotional regulation strategy, will be positively associated with negative personal, social, and psychological consequences.*

**H3:**  *Lower levels of global self-esteem will correlate with higher scores in overall cybersex addiction severity metrics and individual hypersexual behavior dimensions.*

**H4:**  *A longer duration of retention within the specialized therapeutic support program will be associated with lower scores in hypersexual impulse control difficulties, reflecting a descriptive pattern of clinical stability.*

## 2. Materials and Methods

### 2.1. Participants and Sampling

A non-probabilistic convenience sampling strategy was utilized to recruit participants from a single, specialized clinical setting—a dedicated behavioral addiction therapeutic support unit located in the city of Valencia (Spain). Due to the specific nature of this recruitment method and the geographic restriction to a single specialized center, it must be explicitly acknowledged that this study carries an inherent risk of selection bias, which restricts the external validity and generalizability of the findings to the broader population of individuals with cybersex addiction.

The final clinical cohort comprised *n* = 27 participants characterized by a predominantly male distribution (26 males and only one female participant) who met the established inclusion criteria: (a) being aged ≥ 18 years, (b) having a formal clinical assessment indicating high-intensity or problematic cybersex and online pornography consumption, and (c) being actively engaged in the long-term structured support programs of the unit. Regarding the demographic configuration, the sample marked gender asymmetry, consisting of 26 male participants (96.3%) and only one female participant (3.7%). Rather than an intentional design choice, this composition reflects the real-world healthcare demand and treatment-seeking patterns observed in Spanish specialized public and clinical units, where help-seeking behavior for severe cybersex and hypersexuality dysregulation is historically and predominantly driven by male individuals. Consequently, this sample distribution restricts the applicability of the descriptive data exclusively to male clinical profiles, preventing any epidemiological generalization to female populations. Furthermore, the established exclusion criteria were deliberately restrictive, ruling out individuals with active substance use disorders, acute psychotic episodes, severe unmanaged mood disorders, or advanced cognitive impairments. While these strict exclusion criteria significantly optimized the internal validity and clinical homogeneity of the cohort—ensuring that the observed variations in self-esteem and coping strategies were directly linked to cybersex dynamics rather than severe confounding psychiatric conditions—they simultaneously generated a highly selected sample. This restrictive filtering reduces the representativeness of the cohort relative to real-world psychiatric environments, where cybersex addiction frequently presents alongside complex psychiatric comorbidities and dual pathologies.

### 2.2. Procedure

The study procedure was systematically structured into sequential phases to guarantee ethical compliance, clinical safety, and data integrity. The recruitment process took place during the scheduled follow-up or group therapy sessions at the specialized behavioral addiction unit. Potential participants were initially identified by a designated researcher (independent of the direct therapeutic team) who reviewed the unit’s active clinical registry against the established inclusion and exclusion criteria [25].

To mitigate potential therapist bias in participant selection regarding the risk of clinicians selecting only patients with better therapeutic progress or milder symptoms, a consecutive enrollment strategy was implemented. Every single patient who was actively enrolled in the long-term support program and met the objective diagnostic thresholds during the study window was systematically approached. The clinical therapists responsible for the patients’ psychological care had no involvement in the selection design, recruitment interviews, or subsequent data management, thereby establishing a methodological separation between treatment provision and data collection.

Eligible individuals were invited to a private briefing room where the independent researcher explained the study’s scope, objectives, and strict confidentiality protocols. It was emphasized to all candidates that refusal to participate would have absolutely zero impact on their ongoing healthcare or status within the support program. Those who agreed to participate signed a physical Informed Consent form prior to any assessment. Data collection was performed in a quiet, standardized environment within the clinic using pen-and-paper self-report psychometric batteries (ISST, HBI, and RSES). To protect participant privacy and minimize social desirability bias, questionnaires were coded with alphanumeric identifiers, and no personal identifying information was stored. Finally, in strict accordance with the ethical tenets of the Declaration of Helsinki, participation was entirely altruistic; participants received no financial compensation, economic incentives, or clinical privileges of any kind for their time or involvement in the study.

### 2.3. Instruments

Sociodemographic variables [26]—including age, sex, marital status, and employment status—were collected via a customized questionnaire, alongside the following psychometric instruments:

The Internet Sex Screening Test (ISST) was utilized to screen for problematic internet-mediated sexual behavior. This study applied the standard Spanish validation, which preserves the original structural integrity of the instrument. A score of ≥9 out of the total items is conventionally established as the cut-off threshold indicating an established clinical risk of cybersex addiction. While contemporary psychometric literature cautions that this threshold may yield false positives in community convenience samples—frequently due to moral or religious incongruence regarding pornography consumption rather than genuine impulse dysregulation—its diagnostic validity is robust when applied to treatment-seeking clinical cohorts exhibiting objective functional impairment, such as the current sample. In terms of psychometric properties, the Spanish version has demonstrated good reliability, with a reported Cronbach’s alpha ranging between 0.89 and 0.92, confirming its internal consistency for evaluating clinical dimensions of cybersex [27,28].

The Hypersexual Behavior Inventory (HBI) was deployed as a multidimensional tool to assess hypersexuality across three core dimensions: Coping, Control, and Consequences. This study utilized the Spanish-validated version of the scale. Following the guidelines established by its adaptation authors, a total score of ≥53 was used as the cut-off score to classify individuals presenting with clinically significant hypersexual behavior. Although some methodological frameworks debate the specificity of the HBI ≥53 threshold in general population surveys due to its vulnerability to over-pathologizing normative sexual expressions, its diagnostic accuracy is widely endorsed for clinical routing and treatment monitoring in specialized units. The psychometric validation of the Spanish HBI displays adequate performance, with a global Cronbach’s alpha of 0.93, and individual subscale coefficients exceeding 0.85 (Coping: α = 0.89; Control: α = 0.87; Consequences: α = 0.86), establishing it as a dependable metric for psychiatric and mental health nursing assessments [29,30,31].

The Rosenberg Self-Esteem Scale (RSES) was utilized for the evaluation of global self-esteem. It consists of 10 items (five positive and five negative statements) scored on a scale from 1 to 4, yielding a total score range of 10 to 40 points [32]. The original authors did not establish normative cut-off points; consequently, various studies have proposed thresholds based on the distribution of the scale within specific populations. Within the context of Spanish literature, diverse studies have utilized similar cut-off points to identify low levels of self-esteem. The Spanish version of the RSES has demonstrated good psychometric properties, commonly exhibiting an internal consistency greater than 0.80 in adult populations [33].

### 2.4. Study Variables

The study variables were structured according to their clinical and chronological nature. The primary chronological and exposure variables considered were the chronological age of the participants, the age of onset of pornography consumption, and the duration of permanence in the active treatment program, measured in months. Conversely, the clinical outcome variables consisted of the global scores and specific dimensions of the administered screening instruments: for the ISST, the facets of compulsivity, social behavior, solitary behavior, expenditure, and perception of severity were analyzed, whereas for the HBI, the dimensions of coping, control, and consequences were evaluated. This categorization facilitated the analysis of the concurrent associations between the precocity of consumption, program retention duration, and the current severity of hypersexual symptomatology.

### 2.5. Statistical Analysis

Given the specific nature of this clinical cohort and the difficulty of accessing actively treating population segments, the final sample size was established at *n* = 27. It must be explicitly acknowledged that this sample size constrains the overall statistical power of the non-parametric analyses performed (Spearman’s rank correlations and Mann–Whitney U tests), thereby increasing the mathematical probability of incurring Type II errors defined as the failure to detect an actual effect or association due to limited power. To facilitate clinical visualization and provide a descriptive profiling of the cohort, certain continuous variables (such as treatment duration and chronological age) were dichotomized as an auxiliary step using median splits to perform subgroup comparisons via Mann–Whitney U tests. It must be explicitly acknowledged that the artificial dichotomization of continuous metrics inherently leads to a loss of statistical power, information density, and variance. Therefore, these subgroup comparisons were treated strictly as secondary, descriptive segmentations optimized for clinical readability. To prevent any loss of critical data, continuous analyses using direct non-parametric Spearman rank correlations (ρ) were strictly prioritized and maintained as the primary mathematical core for evaluating monotonic relationships among all study variables.

Given the exploratory nature of this study, the multiple correlation matrices were evaluated without applying conservative adjustments for multiple comparisons (such as the Bonferroni correction). This deliberate statistical approach was chosen to maximize the sensitivity for detecting preliminary clinical trends within a hard-to-reach population segment, acknowledging that it simultaneously inflates the risk of Type I errors. Consequently, all computed Spearman correlations must be interpreted strictly as an exploratory data screening tool aimed at generating clinical hypotheses for future fully powered trials, rather than definitive inferential proof.

Following the statistical recommendations of the American Psychological Association (APA) and contemporary biostatistical frameworks, effect sizes were calculated and reported systematically for all statistical tests conducted throughout the study, completely independent of their associated *p*-values. This uniform approach ensures that the interpretation of clinical magnitude is not selectively restricted to marginal or significant results. For continuous bivariate relationships, Spearman’s rank correlation coefficients (ρ) served directly as the standardized effect size metric. For non-parametric subgroup comparisons using the Mann–Whitney U test, the rank-biserial correlation coefficient (r) was systematically computed as the effect size index. Both metrics were uniformly interpreted according to Cohen’s established thresholds: values around 0.10 indicate a small effect size, around 0.30 reflect a medium/moderate clinical magnitude, and values equal to or greater than 0.50 represent a large clinical effect size [34,35,36,37,38].

### 2.6. Ethical Considerations

This study adhered to the principles of the Helsinki Declaration [39] and was approved by the Research Ethics Committee of the Catholic University of Valencia (procedure number UCV/2022-2023/176). Patients included in the study provided informed consent after being informed of the study’s characteristics. The participants were informed that there would be no harmful effects.

## 3. Results

### 3.1. Descriptive Statistics

The study included a clinical sample consisting of 27 participants. The predominant profile was male (96.3%) and professionally active (85.2%). Regarding marital status, a balanced distribution between single (48.1%) and married (51.9%) individuals was observed. The identified consumption pattern exhibited a high frequency, with 92.6% of the sample consuming pornography at least once a day (51.9% daily and 40.7% multiple times a day) (Table 1).

Regarding the consumption history, an early onset of exposure to pornography was identified, with the mean situated in early adolescence (*M* = 13.56 years, *SD* = 4.90; minimum and maximum values of 7 and 35 years, respectively). The recurrent consumption pattern was established, on average, at 18.00 years of age (*SD* = 6.47). Regarding the duration of the consumption sessions, the descriptive analysis revealed an average of 58.70 min (*SD* = 41.52), reaching up to 180 min per session in some subjects (Table 2).

Regarding the screening instruments (Table 3), the severity of cybersex addiction (ISST) obtained a mean of 11.22 (*SD* = 4.29), exceeding the clinical threshold as a group baseline. Conversely, hypersexual behavior (HBI) showed a mean score of 43.15 (*SD* = 14.50), which situates the overall cohort mean below the established clinical cutoff. Upon categorizing the symptomatology using the validated cut-off points, it was observed that 40.7% of the patients (*n* = 11) exceeded the ISST clinical threshold (≥9), and 22.2% (*n* = 6) exceeded the HBI clinical severity threshold (≥53). These data indicate that a portion of the cohort scored above the clinical thresholds for hypersexuality and cybersex addiction. Finally, the mean score for self-esteem was 25.85 (*SD* = 5.23), a value situated at the lower limit of the ‘average self-esteem’ category. This finding is consistent with the prevalence of low self-esteem identified in the frequency analysis (48.1%), reflecting the clinical distribution of self-evaluation scores within this cohort.

### 3.2. Correlation Between Age of Onset and Severity (HBI)

To evaluate the primary clinical associations, the distribution of the data was initially assessed via normality tests, confirming a non-normal distribution for the HBI global scores. Consequently, non-parametric analyses were applied. The association between the age of consumption onset and current severity was evaluated, and the results did not reveal a statistically significant association (ρ = −0.125, *p* = 0.536). This pattern suggests that the precocity of the first contact with pornography demonstrates no linear correlation with the intensity of hypersexual behavior in adulthood within this cohort.

Additionally, the sample was dichotomized using the median age of onset (12 years) to perform a rank comparison. The analyses confirmed the absence of significant differences in overall severity (*p* = 0.489) and impulse control (*p* = 0.322) between subjects with childhood onset (≤12 years) and those with a later onset.

### 3.3. Relationship Between Self-Esteem and Behavior Severity

The correlational analysis did not reveal significant links between self-esteem levels and addiction severity measured by the HBI (ρ = −0.198, *p* = 0.261) or behavior severity measured by the ISST (ρ = −0.063, *p* = 0.722). Although 48.1% of the sample presented clinically low levels of self-esteem, this variable functions as a concurrent comorbidity and demonstrates no statistically significant relationship with the intensity of the addictive symptomatology under the current sample size constraints.

### 3.4. Impact of Treatment Duration on Clinical Severity

The relationship between the time spent in the support program (measured in months) and the severity of hypersexual behavior (HBI_Total) was explored. The correlational analysis showed a moderate negative coefficient (ρ = −0.315); however, this association did not achieve statistical significance (*p* = 0.109), precluding the confirmation of a statistically significant correlation within this sample. While this prevents drawing statistical conclusions, from a purely descriptive standpoint, the direction of the coefficient highlights an exploratory trend toward lower symptomatology among participants with longer treatment durations. This lack of statistical significance, contrasted with the descriptive trend, is likely constrained by the limited statistical power of our sample size (*n* = 27), which restricts the detection of subtle statistical effects.

Similarly, when analyzing the relationship between the duration of therapeutic support and overall cybersex addiction severity via the ISST, the analysis revealed a moderate negative coefficient (ρ = −0.315); however, this association was not statistically significant (*p* = 0.109). Due to the lack of strict statistical significance and the exploratory configuration of the study (unadjusted for multiple comparisons), a definitive clinical link between treatment longevity and symptom reduction cannot be inferred from these data. This result stands as a non-conclusive, descriptive baseline trend that requires extreme caution in its interpretation, representing an exploratory pattern that remains to be tested in larger, confirmatory sample sizes.

**Figure 1:**
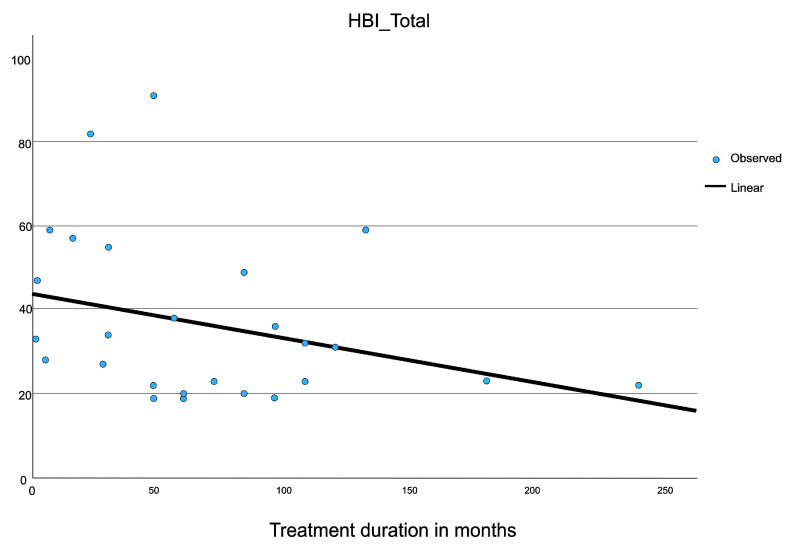
Linear estimation model between treatment time and the hypersexual behavior index (HBI).

The curve estimation analysis for the HBI_Total variable as a function of treatment duration (Figure 1) exhibits a constant negative slope. This graphical representation demonstrates that a longer duration in therapeutic support co-occurs with lower descriptive symptom severity, observing lower hypersexuality scores in those subjects with a more prolonged permanence in the group at the time of evaluation.

### 3.5. Interdependence Between Clinical Dimensions and Therapeutic Stability Factors

As an auxiliary descriptive step to visualize potential behavioral differences within the cohort, the sample was segmented using median splits. While acknowledging the mathematical limitations and loss of power inherent to variable dichotomization, this grouping allows for a preliminary, descriptive screening of sub-profiles. To compare these sub-profiles, Mann–Whitney U tests were conducted. Additionally, a statistically significant positive correlation was found between age and the time spent in the program (ρ = 0.607, *p* < 0.001), indicating that older patients within this cohort had longer program retention.

Likewise, financial expenditure was significantly linked to social interactive behavior (ρ = 0.666, *p* < 0.001) and not to solitary consumption.

**Figure 2:**
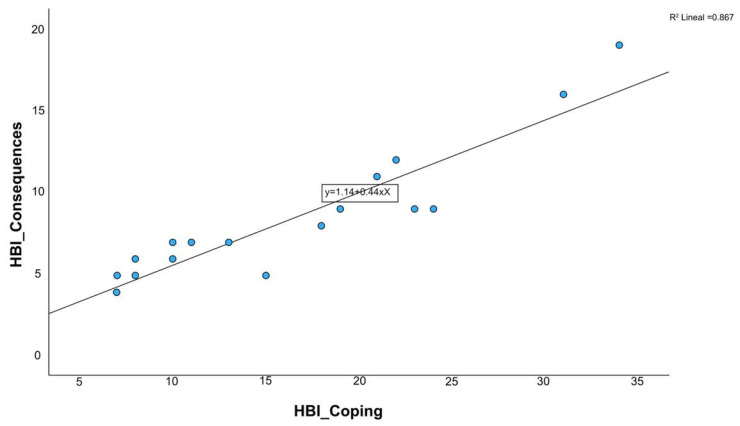
Linear correlation (ρ = 0.917; R^2^ = 0.867) between the Coping and Consequences dimensions of the HBI.

Regarding impulse control scores, the correlation between the Control dimension (HBI) and therapeutic support duration did not reach statistical significance (ρ = −0.350, *p* = 0.073). Similarly, when dichotomizing the group, the Mann–Whitney U test indicated the absence of statistically significant differences (Z = −1.755, *p* = 0.079). Nevertheless, to distinguish between strict significance and practical magnitude, the effect size was calculated, yielding a rank-biserial correlation of r = 0.33. Following Cohen’s criteria [38], this represents a moderate effect size. Therefore, while the data lack the statistical power to declare a significant difference, they offer preliminary descriptive evidence of an exploratory trend regarding impulse control that warrants further exploration in larger sample sizes (Figure 2).

## 4. Discussion

The objective of the present study was to characterize the sociodemographic and clinical profile of a cohort of patients with hypersexual behavior, evaluating the associations between treatment duration, related clinical factors, and symptom severity.

### 4.1. Precocity and Clinical Severity

The principal finding of this study indicates that, within a sample characterized by high-intensity consumption, the age of onset does not act as a linear predictor of severity in adulthood. Although the mean age of first contact with pornography occurs during early adolescence (*M* = 13.56 years), these data align with literature describing early exposure in adolescent populations and its potential association with subsequent problematic pornography use and hypersexual behaviors [40]. However, within this cohort, the correlational analysis demonstrated the absence of a significant association between these factors (ρ = −0.125), which questions the value of onset as an isolated descriptive indicator in chronically affected patients.

In contrast to this lack of statistical significance regarding the age of onset, the meaningful positive correlation between chronological age and treatment duration is notable (ρ = 0.607; *p* < 0.001), suggesting that biological maturity is positively associated with program adherence. The literature on behavioral addictions indicates that factors such as motivation for change, cognitive risk appraisal, and mature social support are associated with stable treatment trajectories and lower dropout rates [41].

This result contrasts with a portion of the literature that links early onset to greater dysregulation of the sexual impulse, suggesting that, in cohorts with already established and long-standing addictions, other proximal clinical factors—such as coping mechanisms—acquire greater clinical relevance than age of onset alone [42,43].

Nevertheless, recent studies [40,43] support this perspective, indicating that the transition toward problematic use is primarily associated with attempts to regulate negative affective states and compensate for psychological vulnerabilities, rather than the precocity of consumption. Hypersexual behavior frequently emerges as a maladaptive strategy to manage negative affective states. From an observational standpoint, these data indicate that the persistent use of online sexual content is associated with an ongoing attempt to regulate emotional distress through behavioral avoidance, which relates to the maintenance of the addictive patterns in adulthood, independent of the age of onset.

Conversely, it is necessary to contextualize these findings within the broader context of behavioral addictions. Extensive literature focusing on community, non-clinical, or less severe samples consistently identifies early onset as a robust correlate of subsequent symptom severity. In non-treatment-seeking individuals, early exposure often relates to progressive sensitization of the reward system, which is associated with problematic use.

However, the lack of statistically significant correlation (ρ = −0.125) highlights a potential phenomenological difference between community samples and chronic, treatment-seeking cohorts. Within this clinical cohort, the initial age of exposure does not demonstrate associative weight, concurrent with the presence of proximal maintenance mechanisms such as structural neural adaptations, maladaptive coping strategies, and interpersonal difficulties.

### 4.2. The Transversal Role of Self-Esteem

A notable clinical finding is the high prevalence of identified low self-esteem (48.1%), consistent with studies describing reduced levels of self-esteem and psychological well-being in individuals with hypersexuality or excessive sexual interest [44,45,46]. However, consistent with the findings regarding the age of onset, self-esteem in our sample did not show a significant correlation with hypersexuality scores on the HBI (ρ = −0.198) or the ISST (ρ = −0.063), which questions its role as a primary correlate of symptom severity. This pattern is consistent with models that understand self-esteem as a marker of general psychological distress and emotional vulnerability, rather than as a specific determinant of hypersexual symptomatology [45].

These findings suggest that self-esteem may function not as a direct indicator, but as a concurrent comorbidity or an outcome associated with the psychosocial impact of the disorder. Several studies have described cycles of shame, guilt, and fluctuations in self-esteem in individuals with compulsive sexual behavior, wherein subjective distress is associated with compulsive sexual behaviors as a maladaptive coping mechanism [45].

This clinical association aligns with the findings of Gulczyńska [40] and Van Tuijl et al. [46], indicating that the functional deterioration reported by these patients relates to the psychological burden of these maladaptive coping dynamics. The statistically significant correlation identified between the Coping and Consequences dimensions (ρ = 0.917; R^2^ = 0.867) warrants a deeper psychometric and conceptual evaluation. This statistical phenomenon can be explained through a reciprocal psychopathological pattern: for an individual with established cybersex addiction, the act of utilizing sexual behavior as an urgent, maladaptive coping mechanism to regulate negative affect (*Coping*) is inherently and inherently linked to catastrophic psychosocial, occupational, or relational consequences. At this threshold of severity, this mechanism co-occurs with immediate negative outcomes, including subjective distress and functional impairment. Therefore, while the subscales remain conceptually distinct in theory, they function as a highly integrated pathological entity in high-severity clinical realities, a nuance that future psychometric adaptations for clinical cohorts should address.

From a strict methodological perspective, a coefficient of this magnitude suggests a high degree of conceptual overlap or potential multicollinearity within the HBI when applied to this specific cohort. In standard validation studies of the HBI using community or broader online samples, these subscales typically demonstrate sufficient statistical independence to justify a three-factor structure. However, our findings indicate that in advanced clinical cohorts characterized by established severity and high-intensity consumption (92.6% daily or multi-daily use), the empirical distinction between these constructs is significantly reduced.

This finding warrants careful contrast with wider epidemiological research. In non-clinical populations and early-stage users, low self-esteem is frequently documented as a primary vulnerability factor associated with hypersexual behavior, acting as an emotional state linked to the search for online sexual gratification. In our clinical sample, however, the absence of a direct correlation suggests that once a severe behavioral addiction is consolidated, self-esteem functions as a concurrent comorbidity rather than a linear correlate. At this established severity threshold, the psychological distress and the erosion of the self-concept are generalized across the entire cohort (as evidenced by the 48.1% prevalence of low self-esteem), effectively creating a ceiling effect that flattens any linear correlation with current symptomatic severity.

### 4.3. Support and Therapeutic Adherence

The observed difference in the mean ranks of the HBI (from 16.57 to 11.23) between groups suggests that impulse control (which did not reach statistical significance, *p* = 0.079) requires careful evaluation. This descriptive pattern aligns with the results of psychodynamic group programs and relapse prevention for compulsive sexual behavior, where outcomes are often associated with high treatment adherence [41]. When critically examining the role of treatment duration within this clinical cohort, the findings must be interpreted with strict methodological caution. The analysis revealed that the negative correlation between retention time in the support unit and overall hypersexuality indicators failed to reach conventional statistical significance (ρ = −0.315, *p* = 0.109). Given the cross-sectional nature of our design, it is fundamentally impossible to infer therapeutic efficacy or any causal relationship between the support program and symptom reduction. A cross-sectional study offers a concurrent assessment of variables at a single point in time, preventing the establishment of chronological sequences, prospective timelines, or longitudinal effects. Consequently, these data do not prove that long-term support causes stabilization, but rather describe a pattern where prolonged clinical retention concurrently coexists with higher metrics of perceived behavioral control [42].

By integrating these findings, an exploratory pattern of symptomatic stabilization emerges: the duration of support programs shows a clinically relevant baseline trend in impulse control, reflecting a potential clinical pathway where prolonged retention aligns with lower levels of addictive behavior [43,44].

**Figure 3:**
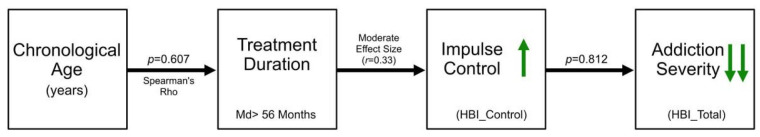
Diagram of concurrent exploratory associations and observed clinical trends within the cohort. Note. The pathways and connectors displayed in this diagram represent the structure of concurrent statistical associations and exploratory trends observed within our cross-sectional design. Given the observational and strictly cross-sectional nature of the study, these lines do not imply a chronological sequence, predictive mediation, or causal pathways among the analyzed variables.

This conceptual mapping of observed associations, represented in Figure 3, reinforces the clinical convergence of prolonged support. It suggests that while chronological age correlates with higher group retention, longer retention concurrently aligns with lower metrics of impulse dysregulation and overall severity. Consequently, this network of concurrent relationships provides a comprehensive overview of the clinical status of chronic cohorts under treatment.

The diagram illustrates the interconnected statistical patterns observed within the cohort. Chronological age is significantly correlated with a longer treatment duration (ρ = 0.607, *p* < 0.001). In turn, longer program retention exhibits a descriptive, moderate clinical effect size (r = 0.33) in relation to better impulse control scores. Finally, impulse control displays a statistically significant correlation with overall addiction severity metrics. The lines represent concurrent statistical associations observed in our cross-sectional design; they do not imply a causal sequence, predictive mediation, or multi-step inferential pathways.

Consequently, we must emphasize that these findings represent exploratory clinical hypotheses rather than confirmed causal pathways, given the lack of strict statistical significance. The fact that a moderate clinical effect size (r = 0.33) was observed alongside a non-significant *p*-value statistically implies that our study was underpowered. Therefore, these data should not be read as evidence of efficacy, but as an indication that the relationship between support duration and impulse control is a clinically relevant phenomenon that remains to be tested robustly through high-powered longitudinal designs. Additionally, no significant differences were identified in the severity of cybersex addiction measured by the ISST (*p* = 0.751), suggesting a differential distribution of scores depending on the evaluated psychometric construct.

### 4.4. Limitations of the Study

Despite the clinical relevance of characterizing a specialized help-seeking cohort, this research presents methodological limitations that necessitate a highly cautious interpretation of the findings and directly affect the internal and external validity of our conclusions.

First and foremost, the cross-sectional nature of the design constitutes a limitation to internal validity, as it precludes the establishment of direct causal relationships between the analyzed variables. Specifically, the observed association between longer therapeutic support (tenure > 56 months) and lower hypersexual severity (HBI scores) must be critically evaluated under the scope of survivor bias. Because we did not employ a longitudinal design with baseline measures, alternative explanations for this pattern cannot be ruled out. It is possible that the long-term subgroup does not exhibit lower severity as a direct cumulative consequence of the intervention but rather represents a highly selective subpopulation. In clinical settings for behavioral addictions, patients with greater baseline psychopathology, lower impulse control, or poorer social support are associated with higher rates of premature therapeutic dropout. Consequently, our long-term sample might be artificially composed only of ‘surviving’ patients who possessed a more favorable intrinsic prognosis or milder baseline clinical profiles from the beginning.

Furthermore, the limited sample size severely restricts the statistical power of our inferential analyses. While the sample’s exceptional clinical homogeneity—ensured by strict psychiatric exclusion criteria—and the extreme intensity of their consumption patterns grant the study strong internal validity for this specific profile, the small sample size increases the risk of Type II errors. For instance, the moderate negative correlation between treatment duration and overall hypersexuality severity (ρ = −0.315) failed to reach strict statistical significance (*p* = 0.109). Although our calculated moderate effect size (r = 0.33) suggests a clinically meaningful pattern, the low statistical power prevents us from confirming whether this relationship is robust or a statistical artifact.

Another salient methodological limitation involves the potential presence of social desirability bias, which was not explicitly addressed in our initial framework. Cybersex addiction and hypersexual behaviors are intrinsically linked to social stigma, moral incongruence, and profound feelings of shame.

Because the diagnostic and psychometric questionnaires were administered face-to-face by healthcare professionals within the active therapeutic environment, participants may have sub-consciously or consciously altered their responses. Specifically, patients might have underreported the current severity of their consequences or overstated their adaptive coping strategies to present a more favorable image of recovery or to align with the perceived expectations of the clinical staff (the ‘good patient’ effect).

While we attempted to minimize this bias by strictly ensuring data blinding, processing the scores exclusively for aggregated statistical research, and leveraging the strong pre-existing therapeutic alliance characterized by non-judgmental acceptance, the influence of social desirability cannot be fully controlled. This contextual factor requires that the self-reported scores, particularly the high scores on the HBI Coping and Consequences subscales, be interpreted with appropriate clinical caution, as they may reflect a combination of authentic psychological distress and socially conditioned reporting.

Finally, limitations regarding external validity restrict the generalizability of our proposed mapping of concurrent associations. The overwhelming male representation (96.3%) and high employment rate (85.2%) accurately mirror the specific healthcare demands within specialized behavioral addiction units in Spain, but limit the generalizability of how these dynamics operate in female populations or different socioeconomic contexts. Women experiencing cybersex addiction frequently display distinct consumption patterns, different preferred platforms, and alternative emotional coping mechanisms that were not evaluated in this study. Therefore, our final model must be strictly interpreted as a preliminary clinical characterization of male patients undergoing long-term treatment settings, rather than a universal framework for hypersexuality recovery. Rather than merely replicating general population trends, this study offers a distinct contribution by psychometrically and sociodemographically characterizing an understudied clinical population: individuals with severe, long-standing cybersex addiction embedded in long-term specialized therapeutic programs (>56 months).

The core novelty of our findings lies in demonstrating distinct psychopathological patterns within clinical cohorts experiencing advanced behavioral addiction severity. In this cohort, traditional baseline predictors—such as early onset or low self-esteem—lose their linear predictive value for current symptom severity. Instead, they operate as concurrent comorbidities (evidenced by the 48.1% prevalence of low self-esteem and the strong correlation between coping and consequences). This suggests that established clinical severity is associated with a clinical distribution that characterizes how these variables present, an observation previously not identified in broader community or non-treatment-seeking samples.

### 4.5. Actionable Implications for Clinical Practice

These findings translate into several concrete guidelines for specialized healthcare professionals and mental health nurses managing advanced cybersex addiction:

1. Re-evaluation in Assessment Paradigms: Clinicians should not rely solely on traditional hypersexuality severity scores (like total HBI) to evaluate clinical progress, as severe functional consequences and maladaptive coping mechanisms co-occur even among patients with prolonged program tenure. Instead, screening should focus on continuous monitoring of daily emotional regulation strategies and associated psychological distress.
2. Intervention Redesign: Because early onset loses its relative weight in advanced clinical severity cohorts, therapeutic efforts in long-term support groups should focus less on reconstructing initial triggers or early exposure history. Resources should instead be directed to managing proximal maintenance mechanisms, specifically advanced distress tolerance and structured cognitive-behavioral coping protocols.
3. Role of Specialized Nursing: The data support the potential integration of long-term nursing-led support structures to manage chronic psychiatric comorbidities and relational difficulties, acting as a continuous support system for patients vulnerable to relapse.

### 4.6. Future Research Directions

To address the limitations of the current study, future research should utilize longitudinal designs. First, prospective longitudinal tracking with multi-center clinical cohorts is necessary to evaluate the long-term patterns of specialized treatment. Second, future designs must explicitly incorporate time-varying covariates to monitor therapeutic dropouts, which are required to control for survivor bias. Finally, expanding the recruitment criteria to include female patients and diverse socioeconomic backgrounds is important in order to examine whether the observed associative patterns identified here are applicable to other populations or specific to specialized male treatment-seeking populations.

In summary, while the insights gained from this cohort provide a valuable and necessary clinical characterization of long-term cybersex addiction management, the interpretation of these patterns must be considered within the framework of our study design. Due to the cross-sectional design, a small sample size, and an almost exclusively male representation, these findings do not constitute confirmation of therapeutic efficacy or universal generalizability. Instead, they deliver meaningful, descriptive preliminary evidence. They should be considered an initial reference that highlights the potential utility of long-term mental health nursing support, serving to generate specific, testable hypotheses for future, highly powered, multi-center longitudinal studies.

## 5. Conclusions

This study provides a preliminary, highly specialized clinical and sociodemographic characterization of a specific cohort of male patients seeking treatment for cybersex addiction within a specialized healthcare framework. Our findings highlight that within this established clinical severity profile, psychological vulnerabilities such as low self-esteem and functional consequences operate as concurrent comorbidities that remain active regardless of observed psychometric severity patterns.

Regarding therapeutic duration, the identified associations suggest a potential exploratory trend toward lower hypersexual severity in patients associated with extended program tenure. However, due to the study’s cross-sectional design, small sample size, and the inherent risk of survivor bias, these observations cannot be interpreted as definitive proof of causality or long-term treatment efficacy.

Consequently, the prominent role of nursing-led interventions highlighted in this context should not be taken as a definitive clinical mandate, but rather as a preliminary hypothesis. These specialized nursing-led support structures appear to offer a potential framework for managing the concurrent psychiatric comorbidities and maladaptive coping strategies typical of these patients. Nevertheless, before these long-term nursing interventions can be firmly recommended or standardized, further robust, prospective, and longitudinal research with larger, multi-center clinical samples is strictly required to confirm their direct clinical benefits and cost-effectiveness.

## Figures and Tables

**Table 1:** Sociodemographic and consumption characteristics of the sample (*n* = 27).

Variable	Category	*n*	%
Sex	Male	26	96.3
	Female	1	3.7
Marital Status	Single	13	48.1
	Married	14	51.9
Employment Status	Student	3	11.1
	Employed	23	85.2
	Not studying or working	1	3.7
Frequency of Consumption	Weekly	2	7.4
	Daily	14	51.9
	Multiple times a day	11	40.7

**Table 2:** Descriptive statistics of the sociodemographic variables.

Variable	M	SD
Chronological Age	34.00	8.64
Age of Onset Consumption	13.56	4.90
Age of Recurrent Consumption	18.00	6.47
Minutes per Session	58.70	41.52

M = mean; SD = standard deviation.

**Table 3:** Descriptive statistics and categorization of clinical severity in the cohort.

Instrument/Variable	M/*n*	SD/%
**ISST**		
Total Score	11.22	4.29
Low/Moderate Severity (<9)	16	59.3%
High Risk/Problematic Use (≥9)	11	40.7%
**HBI**		
Total Score	43.15	14.50
Dimension: Impulse Control	16.22	8.532
Dimension: Coping	13.37	7.948
Dimension: Consequences	7.04	3.767
Low/Moderate Severity (<53)	21	77.8%
Clinical Severity (≥53)	6	22.2%
**Rosenberg Scale**		
Total Score	25.85	5.23
Low Self-Esteem	13	48.1%

M = mean; SD = standard deviation.

## Data Availability

The data presented in this study are available on request from the corresponding author due to the sensitive nature of the clinical data regarding individuals with cybersex addiction and to protect participant privacy.
